# A hybrid dependency-based approach for Urdu sentiment analysis

**DOI:** 10.1038/s41598-023-48817-8

**Published:** 2023-12-12

**Authors:** Urooba Sehar, Summrina Kanwal, Nasser I. Allheeib, Sultan Almari, Faiza Khan, Kia Dashtipur, Mandar Gogate, Osama A. Khashan

**Affiliations:** 1 Capital University of Science & Technology, Islamabad, 44000 Pakistan; 2 Division of Theoretical Computer Science, KTH Royal Institute of Technology Stockholm, Stockholm, Sweden; 3 Center of Applied Intelligence Systems Research, Halmstad University, 302 50 Halmstad, Sweden; 4 Department of Information Systems, College of Computer and Information Sciences, King Saud University, P.O. Box 51178, Riyadh, Saudi Arabia; 5 Department of Computing and Informatics, Saudi Electronic University, 11673 Riyadh, Saudi Arabia; 6 Riphah International University, Islamabad, 45320 Pakistan; 7 School of Computing, Edinburgh Napier University, Edinburgh, EH10 5DT UK; 8 Research and Innovation Centers, Rabdan Academy, P.O. Box 114646, Abu Dhabi, United Arab Emirates

**Keywords:** Computer science, Information technology

## Abstract

In the digital age, social media has emerged as a significant platform, generating a vast amount of raw data daily. This data reflects the opinions of individuals from diverse backgrounds, races, cultures, and age groups, spanning a wide range of topics. Businesses can leverage this data to extract valuable insights, improve their services, and effectively reach a broader audience based on users’ expressed opinions on social media platforms. To harness the potential of this extensive and unstructured data, a deep understanding of Natural Language Processing (NLP) is crucial. Existing approaches for sentiment analysis (SA) often rely on word co-occurrence frequencies, which prove inefficient in practical scenarios. Identifying this research gap, this paper presents a framework for concept-level sentiment analysis, aiming to enhance the accuracy of sentiment analysis (SA). A comprehensive Urdu language dataset was constructed by collecting data from YouTube, consisting of various talks and reviews on topics such as movies, politics, and commercial products. The dataset was further enriched by incorporating language rules and Deep Neural Networks (DNN) to optimize polarity detection. For sentiment analysis, the proposed framework employs predefined rules to trigger sentiment flow from words to concepts, leveraging the dependency relations among different words in a sentence based on Urdu language grammatical rules. In cases where predefined patterns are not triggered, the framework seamlessly switches to its sub-symbolic counterpart, passing the data to the DNN for sentence classification. Experimental results demonstrate that the proposed framework surpasses state-of-the-art approaches, including LSTM, CNN, SVM, LR, and MLP, achieving an improvement of 6–7% on Urdu dataset. In conclusion, this research paper introduces a novel framework for concept-level sentiment analysis of Urdu language data sourced from social media platforms. By combining language rules and DNN, the proposed framework demonstrates superior performance compared to existing methodologies, showcasing its effectiveness in accurately analyzing sentiment in Urdu text data.

## Introduction

In this age of social media and networks, information spread online influences our choices ranging from selecting a movie to watch to daily purchases such as groceries, clothing, and so on, as well as purchasing services related to health and business. It is now common practice to review products and services online after using and purchasing them or after using a business group’s services. E-commerce sites also encourage their users to comment and review about their products after purchase and use so that those reviews can be used later to improve the product quality or the quality of the services being provided, and also to introduce new products based on the needs of the users. Businesses are using social media analytics to improve their services and products according to the choices and opinions of their target audience through different platforms.

Since the amount of data shared on online social platforms is so large and requires a time-consuming process for pre-processing the raw data to extract the required information, it is exceptionally difficult to interpret it without the assistance of an intelligent automated system. If such systems are not built, the data produced in billions and trillions of dollars will be wasted without being used to help businesses and enterprises improve their services, make them better follow the needs of the users, and improve the quality of the products or services they provide to their target users.

SA is the process of analysing raw data containing user opinions and reviews about a variety of products and services in order to automatically interpret the polarity of the user's opinions shared on social media platforms, as well as in the form of reviews, comments on e-commerce websites, and by bloggers on blogging sites^1,2^.

**Figure 1:**
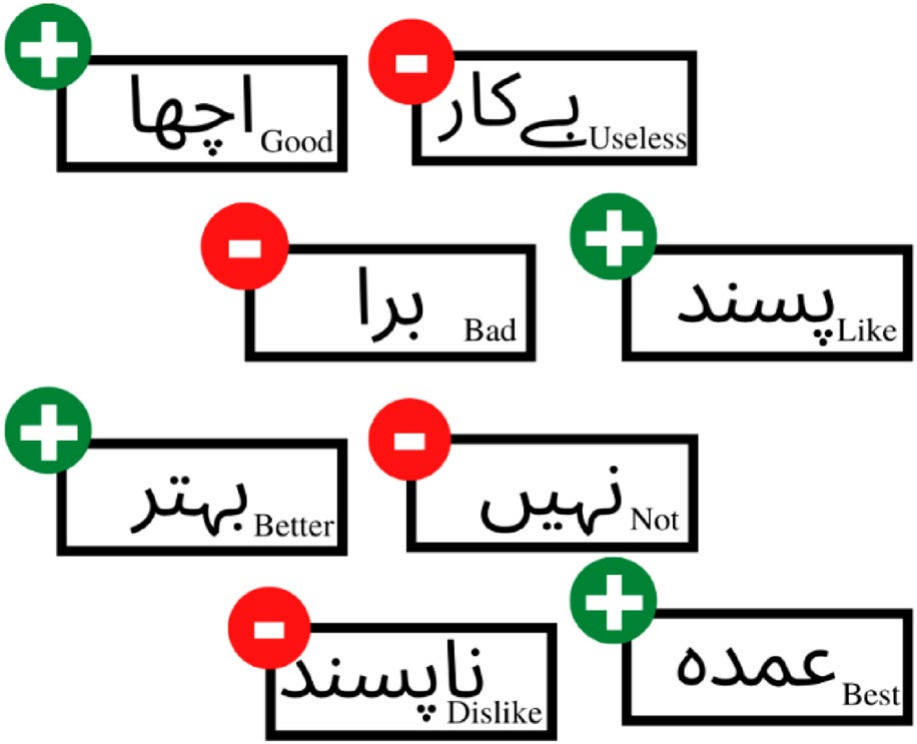
A demonstration of how traditional approaches assign polarity to different words is further used for assigning polarity to a sentence.

Due to the diversity of the languages spoken by people worldwide, SA is a challenging area of research. Technically, the generalisation of proposed models from one language to another is not possible. Even if a model developed for one language is applied to another, significant pre-processing is necessary to reap the benefits. Urdu, as a resource-constrained language, has a long way to go in the field of SA before it can showcase efficient and intelligent SA models. Urdu SA is still in its infancy^3^. Currently, available approaches to SA give scant consideration to dependency-based grammar-based rules. As illustrated in Fig. 1, the majority of models determine the polarity of a complete sentence using the word's co-occurrence polarity, which occasionally fails to correctly classify a sentence according to its grammatical context and the interdependence of different words, which affects the sentence's overall polarity.

Dependency grammar rules are built on patterns of a language that enable the sentiment to move from words to that of the concept relying on the dependency relation between them^4^.

Hence dependency-based rules are considerate of hierarchical relations among different keywords or conjunctions interjections, in order of occurrence of words and polarities of the individual words for a more accurate determination of the underlying polarity of a sentence^5^.

The main objective of this research work is to optimise the detection of sentiment polarity in Urdu sentences containing reviews or opinions on movies, products, and politics by combining Urdu grammar rules with various machine learning (ML) and deep neural network (DNN) models. In this study, a niche framework has been proposed, integrating dependency grammar-based rules for the Urdu language with DL models for the exploration of the Urdu language dataset. The significant contribution of this research paper is as follows:

- Use of grammatical dependency-based rules for Urdu SA.
- A framework that is able to figure out the polarity of Urdu sentences classified based on the individual polarity of words and their correlation and arrangement that is in the rules of Urdu grammar in order to provide a better classification in comparison to the state-of-the-art polarity classification models.
- A comprehensive discussion of different grammatical rules of the Urdu language and how they impact the polarity of sentences.
- The utilisation of the developed Urdu language datasets: movie reviews, political reviews, and product reviews.
- Experimentation by integrating the Urdu language's dependence on grammar rules using models such as Support Vector Machine (SVM), Logistic Regression (LR), and Deep Neural Network (DNN) models such as long short-term memory (LSTM), and Convolutional Neural Networks (CNN).

The structure of this research paper is as follows: section “Literature review” describes the literature review of prior studies on SA approaches, their shortcomings, and challenges. Next, section “Methodology” describes the research methodology and design of proposed study, including the process for collecting the data and proposed framework. Section “A dependency rules-based Sa framework” includes a result and discussion, and comparison of the proposed approach for SA. Finally, section “Dataset” summarises the paper with the conclusion and discusses future endeavours.

## Literature review

In this section, the current research on SA and the use of ML, AI, and DNN in this field has been summarised, along with the research gaps that need to be addressed to make SA systems and approaches more effective and usable by practitioners in various fields.

Subramanian et al.^6^ developed a SA model based on sequence-based Neural Networks, specifically using a CNN-LSTM approach on the IMDB movie review dataset. Alsayat et al.^7^ introduced an ensemble deep learning language model to enhance sentiment analysis in social media applications. By conducting experiments using various datasets, including Twitter's coronavirus hashtag dataset and public review datasets from Amazon and Yelp, they demonstrated that their proposed models outperform other models in terms of classification accuracy. Aljameel et al.^8^ introduced an SA approach for predicting public awareness of COVID-19 prevention measures in Saudi Arabia, using SVM, KNN, NB, and N-gram feature extraction. SVM with bigram in TF-IDF outperformed other models. Rao et al.^9^ utilized multilevel features and a MFCNN model, combining multiple CNN features, to classify English text sentiment, outperforming a conventional CNN model. Yue et al.^10^ proposed a task-oriented, granularity-oriented, and methodology-oriented SA approach for English social media sites. Prottasha et al.^11^ examined the utilization of transfer learning via BERT-based supervised fine-tuning for sentiment analysis (SA). Their findings reveal that incorporating transfer learning and BERT in SA tasks surpasses alternative embedding techniques and algorithms, demonstrating superior performance. Ashir et al.^12^ experimented with SVM, NB, MLP, AdaBoost, and LR classifiers on movie reviews and Twitter samples, reporting accuracy rates of 72% and 91.1%, respectively.

Grammatical rules differ between languages, as does the quality of data and pre-processed data available in that language. Dashtipour et al.^5^ researched SA of hotel reviews in Persian, achieving high accuracy using a hybrid model that combines LSTM with dependency-based grammatical rules. Miranda et al.^13^ conducted a comprehensive study on SA in Spanish, focusing on document-level SA. Can et al.^14^ investigated language-generalized SA models, proposing an RNN-based technique for different languages, including resource-constrained languages. Chen et al.^15^ proposed a lexicon-based approach for SA of Chinese social media posts, and developing a comprehensive process and lexicon algorithm in their study. Poria et al.^16^ proposed a multimodal SA classification approach utilizing deep learning algorithms and discussing challenges in multimodal SA research. Zadeh et al.^17^ presented a framework based on tensor fusion techniques for multimodal SA, achieving high accuracy for textual, visual, and acoustic modalities. Rosas et al.^18^ also presented a method for multimodal SA classification that can possibly be used to determine the sentiments expressed in visual data streams at the utterance level. The results of their experiments on the Multimodal Opinion Utterances Dataset (MOUD) indicate that utterance-level sentiment classification achieved a 74.09 100% accuracy across multiple modalities, including Linguistic, Acoustic, and Visual models. Recently, Li et al.^19^ have suggested a novel SA classifier that combines a two-channel classifier with a neural tensor block. They tested their proposed model on three different standard datasets. The BiERU-lc model achieved a weighted average accuracy of 0.74% and an f1 score of 0.45% in their experimental study using IEMOCAP datasets. Chakravarthi et al.^20^ developed a dataset for SA that includes comments in three Dravidian languages: Tamil, Kannada, and Malayalam English. Their dataset was compiled from user comments on various social media platforms, including YouTube. The study's results showed a weighted average accuracy of 0.68%. Kazmaier et al*. *^21^ introduced various techniques for heterogeneous ensembles for SA in their study and analysed results via experimentation on their dataset. Additionally, they developed a novel model for SA based on ensemble learning of multiple SA approaches. The study's findings indicate that the proposed ensemble technique improved the results of SA on the Twitter data set by approximately 5.53% and for the Yelp data set by 0.43%. Aniello et al.^22^ proposed an aspect-based reference SA model and suggested tools for quantifying opinions and sentiments within sentences.

Social media comments and reviews are being analysed to see how SA can affect businesses Cruz et al.^23^ proposed a model to study the impact of financial accounts on stock market decision-making. Wang et al.^24^ investigated the impact of SA models on fundraising campaigns and the growth of Internet finance. Bueno et al.^25^ proposed a model for SA that makes decisions based on the business context. Aziz et al.^26^ proposed a method for SA of reviews and comments on Roman Urdu eCommerce websites. They created a dataset containing 21,000 records with the assistance of a Kaggle dataset. They conducted experiments on a variety of machine learning and deep neural network-based models and compared them to their proposed approach. The results of their experiment study indicate that their model achieved an accuracy of 82.19% when Sentiment classification was estimated using RANSAC (random sample Consensus). Mukhtar et al.^27^ proposed a model for SA in Urdu using a lexicon. Chandio et al.^28^ developed an SVM-based model for SA of Roman Urdu-based eCommerce reviews, reporting accuracy with their created Urdu dataset. Khan et al.^29^ utilized ML and DNN models to analyse multimodal sentiment in Urdu, with linear regression (LR) outperforming other models. Qureshi et al.^30^ proposed an SA model for Roman Urdu reviews, achieving high accuracy using deep neural network techniques and logistic regression. In previous research^31^ DL for multimodal SA of Urdu was used, achieving high accuracy for polarity prediction.

Li et al.^36^ reported an accuracy of 0.91% when employing CNN with an attention layer and transfer learning for SA on a dataset of roman-urdu texts. Using rule-based machine learning like support vector machine, Naive Bayes, Ada boost, Multilayer Perceptron, Linear Regression and Random Forest and deep learning algorithms like Convolutional Neural Network, Long short-term memory, Bidirectional- Long short-term memory, Gated recurrent units (GRUs), and Bidirectional-GRU), Khan et al.^37^ achieved an F1 score of 81.49%. A study was undertaken by Rehman and Soomro^38^ to analyze the sentiment of Urdu messages obtained from the popular social media platform Twitter. Experiments were conducted by the researchers utilizing various machine learning algorithms within the WEKA platform. It was determined that the SMO algorithm exhibited superior performance in sentiment analysis of tweets written in Urdu (Nastaleeq), while the Random Forest approach produced the most favorable outcomes when applied to Roman Urdu text. Chandio et al.^39^ conducted an experiment in their investigation employing RU-BiLSTM, a deep recurrent architecture. This BiLSTM-based architecture includes both word embedding and an attention mechanism. Their investigation was designed to examine the sentiment expressed in Roman Urdu. The experimental procedures executed by the researchers utilizing two datasets of Roman Urdu yielded positive results. Khan et al.^40^ put forth a novel deep learning framework designed for the purpose of sentiment analysis in Roman Urdu and English dialects. This architecture consists of two layers: a Long Short-Term Memory (LSTM) layer for preserving long-term dependencies and a one-layer Convolutional Neural Network (CNN) model for extracting local features. Multiple machine learning classifiers are provided with the feature maps obtained by the Convolutional Neural Network (CNN) and Long Short-Term Memory (LSTM) models so that the highest level of classification can be attained. The evaluated accuracies of these classifiers against the MDPI, RUSA, RUSA-19, and UCL datasets are 0.904, 0.841, 0.740, and 0.748, respectively. The results suggest that for sentiment analysis in Roman Urdu, the Word2Vec CBOW model and the SVM classifier produce more favourable results. On the contrary, for sentiment analysis specifically targeting the English language, the BERT word embedding, two-layer LSTM, and SVM as a classifier function are considered to be more suitable alternatives. Ahmed et al.^41^ presented the meta-learning ensemble approach in their research, which sought to incorporate deep learning and foundational machine learning models for the Urdu language. The execution of this approach involved the utilization of two levels of meta-classifiers. The ensemble method under consideration integrates the predictions produced by the inter-committee and intra-committee classifiers at two distinct levels. By implementing the suggested technique, the classification accuracy of the baseline deep models is significantly improved, as shown by the results.

In their research, Altaf et al.^42^ employed linguistic variables that are unique to the Urdu language to analyze sentiment at the sentence level. Furthermore, conventional machine learning methodologies were utilized in order to categorize idioms and proverbs. For this objective, the researchers employed a dataset that they had curated. The experimental results indicate that the J48 classifier exhibits a higher level of proficiency in sentiment classification, as evidenced by its 90% accuracy rate and 88% F-measure. Bashir et al.^43^ presented the Urdu Nastalique Emotions Dataset (UNED), an assortment of annotated phrases and paragraphs representing diverse emotions. Additionally, the authors put forth a deep learning (DL) methodology that successfully classified six unique categories of emotions present in the UNED corpus. The results of the experiments indicate that the DL-based model outperforms generic machine learning approaches, as evidenced by its F1 score of 85% on the UNED sentence-based corpus and 50% on the UNED paragraph-based corpus. Khan et al.^44^ this research paper introduces a novel framework that capitalizes on the Cognitive Relationship (CR) between sarcasm and sentiment in order to enhance classification precision.

The dataset compiled by the researchers comprised 7000 tweets composed in standard URU language. Furthermore, experiments were conducted employing a CR-based methodology to classify sarcasm and emotion. Based on their research outcomes, it was concluded that eXtreme Gradient Boosting and Linear Regression exhibit superior performance. The implementation of CR has resulted in a significant improvement of 9.3% in sentiment classification when compared to the stand-alone (SA) method. Furthermore, it has consistently increased by approximately 22% in comparison to the distribution at the outset. Likewise, the implementation of CR for the classification of sarcasm has demonstrated a significant increase of 9.1% in comparison to the SA method, and a considerable enhancement of approximately 23.6% over the initial distribution.

Despite recent advancements, there is a research gap in SA for resource-poor languages like Urdu, particularly in concept-level SA. This research aims to address this gap in Urdu linguistics research.

## Methodology

**Figure 2:**
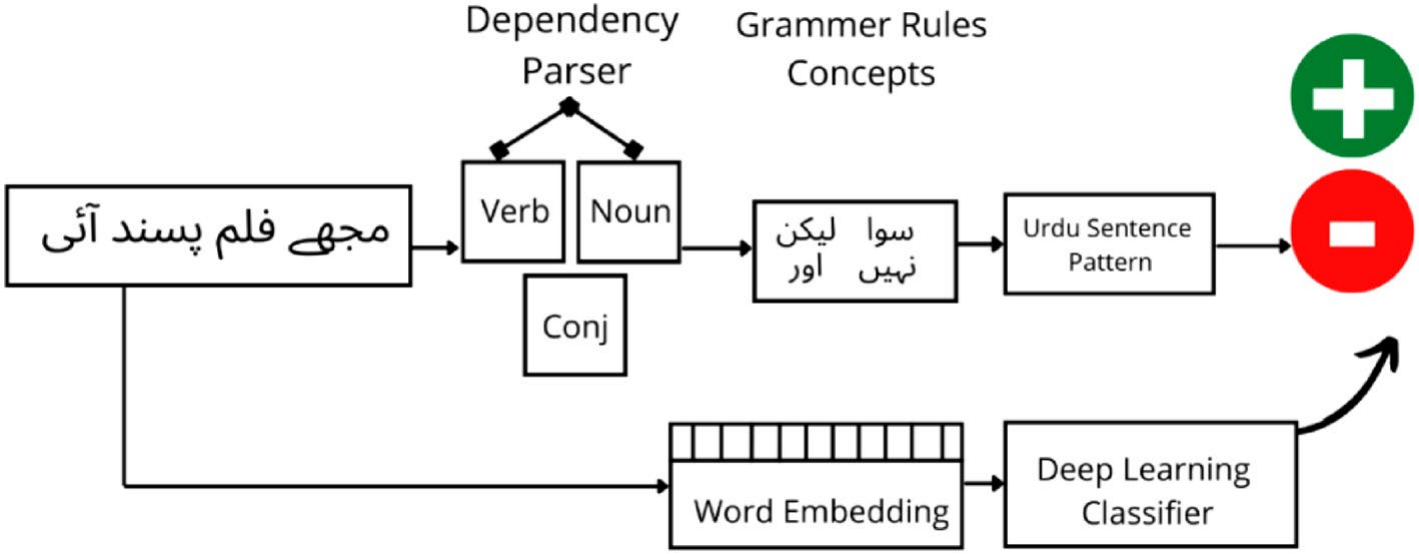
Classification of a sentence polarity based on dependency-based rule example.

In this section, the process of analysing dependency-based rules for Urdu SA has been summarised, as depicted in Fig. 2.

### Identify Urdu grammar rules

**Figure 3:**
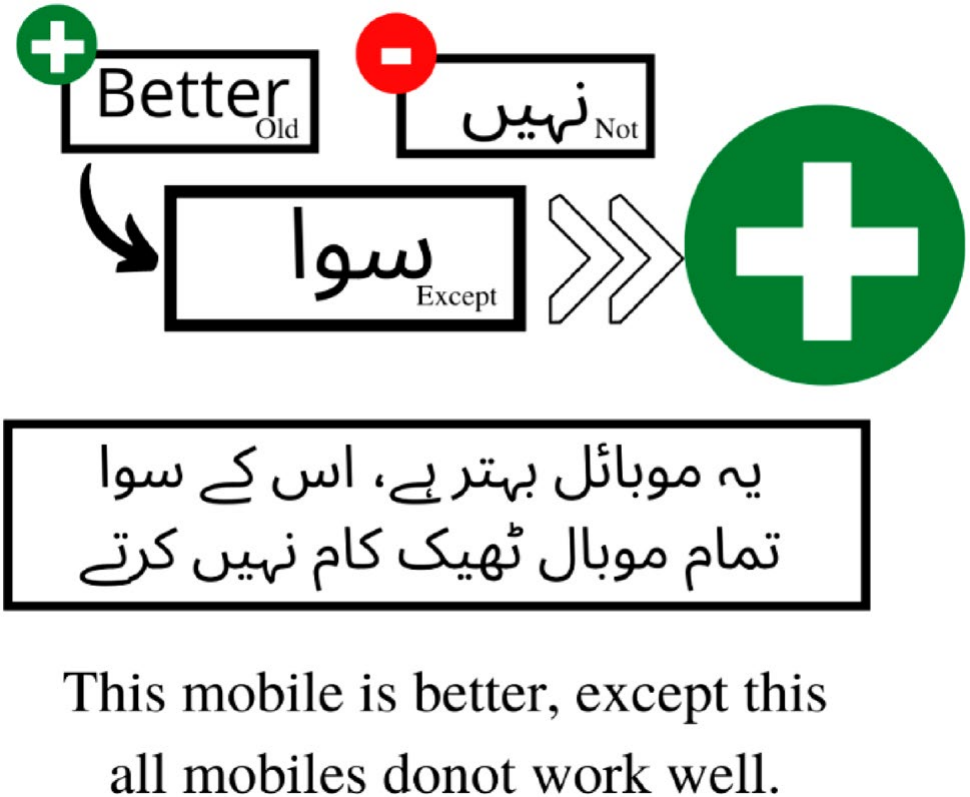
An example of polarity classification of our proposed grammatical rules-based classification technique.

Previous research lacked effective SA due to a failure to consider language rules when assigning polarity to a sentence and instead focusing exclusively on the polarity of individual words. For example, in the sentence demonstrated in Fig. 3, “**یہ موبائل بہتر ہے، اس کے سوا تمام موبال ٹیھک کام نہیں کرتے**” (This mobile is better, rest of them does not work well), looking at the polarity of the words of the sentence due to presence of a word with negative polarity such as ‘نہیں' it seems like the speaker of the sentence has a negative opinion about all mobiles. If this sentence is analysed by a state-of-the-art approach to classifying the polarity of the sentiment, it is possible that this sentence would be categorised as a sentence with negative polarity without considering the context of the sentence. There is also the possibility of having a conflict in the decision of the model as there is also a word with a positive polarity that is “بہتر” “Better”. In such cases, it is not possible to correctly identify the overall polarity of the sentence. Alternatively, considering the grammatical context of the sentence and trying to analyse the real meaning of the sentence keeping in view the dependency-based rules of Urdu grammar, it is a sentence with positive polarity because of the use of the word "سوا”(except). Whenever 'Except' is used in a sentence, it means that word is used for separation in two clauses with mostly opposite polarity. In such cases, the polarity for a single entity that is an exception from another is found in one clause that is before the exception word, and the polarity of the group can be found in the other. So, in our example, the first clause with positive polarity is the deciding factor of the polarity, which could only be possibly identified by the grammatical dependency-based rules of Urdu Grammar.

**Figure 4:**
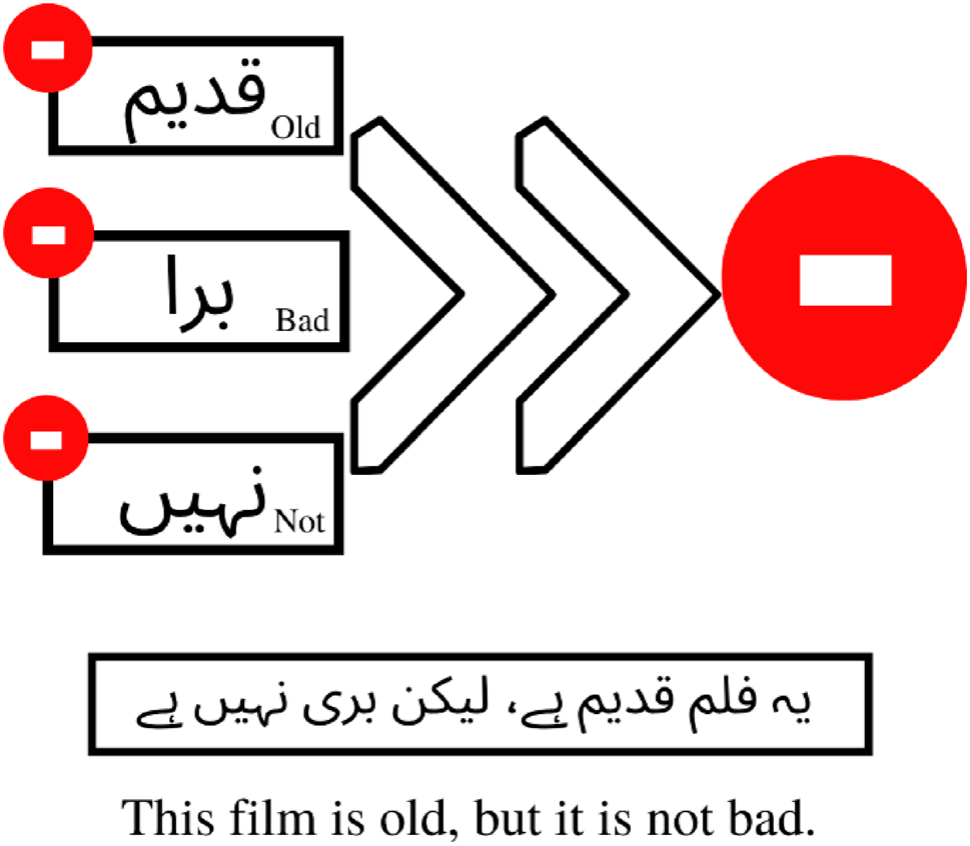
An example of polarity classification of a traditional word-based classification technique.

Consider the relationship between the words in the sentence “**یہ فلم قدیم ہے، لیکن بری نہیں ہے**” (This film is old, but it is not bad). Due to the presence of three words with negative polarity in this sentence, a traditional model will classify it as a sentence with negative polarity, as illustrated in Fig. 4. However, this sentence has a positive polarity in reality due to the presence of the word "But," which shifts the sentence's overall polarity to the positive.

**Figure 5:**
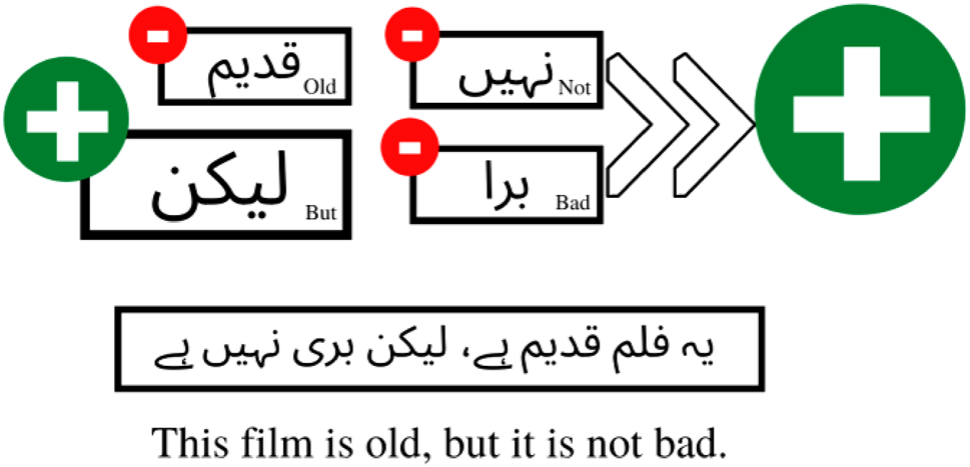
An example of polarity classification of our proposed grammatical rules-based classification technique.

The same sentence would be classified as a positive sentence based on grammatical dependency rules because the word ‘but’ is used to negate the polarity of the first part in the second part. In current case, the negative polarity of ‘This film is old’ is negated by the use of ‘But’ in the sentence. As shown in Fig. 5, two negative polarity words “and” cancel each other out, making the overall polarity of the second part of the sentence positive. Thus, a sentence has positive polarity, which is missed by traditional classification.

As demonstrated by the preceding two examples, it is critical to understand the grammatical context of the language in order to identify the sentence's polarity correctly. The grammatical rules in the Urdu language have been identified that can alter the sentence's polarity in order to construct a model based on those grammatical rules. This research demonstrated how the proposed approach is capable of correctly classifying sentences that cannot be classified using any conventional sentiment classification technique. The following section identifies the various grammatical dependency-based rules that our proposed model for Urdu SA employs.

### URDU grammer rules

**Figure 6:**
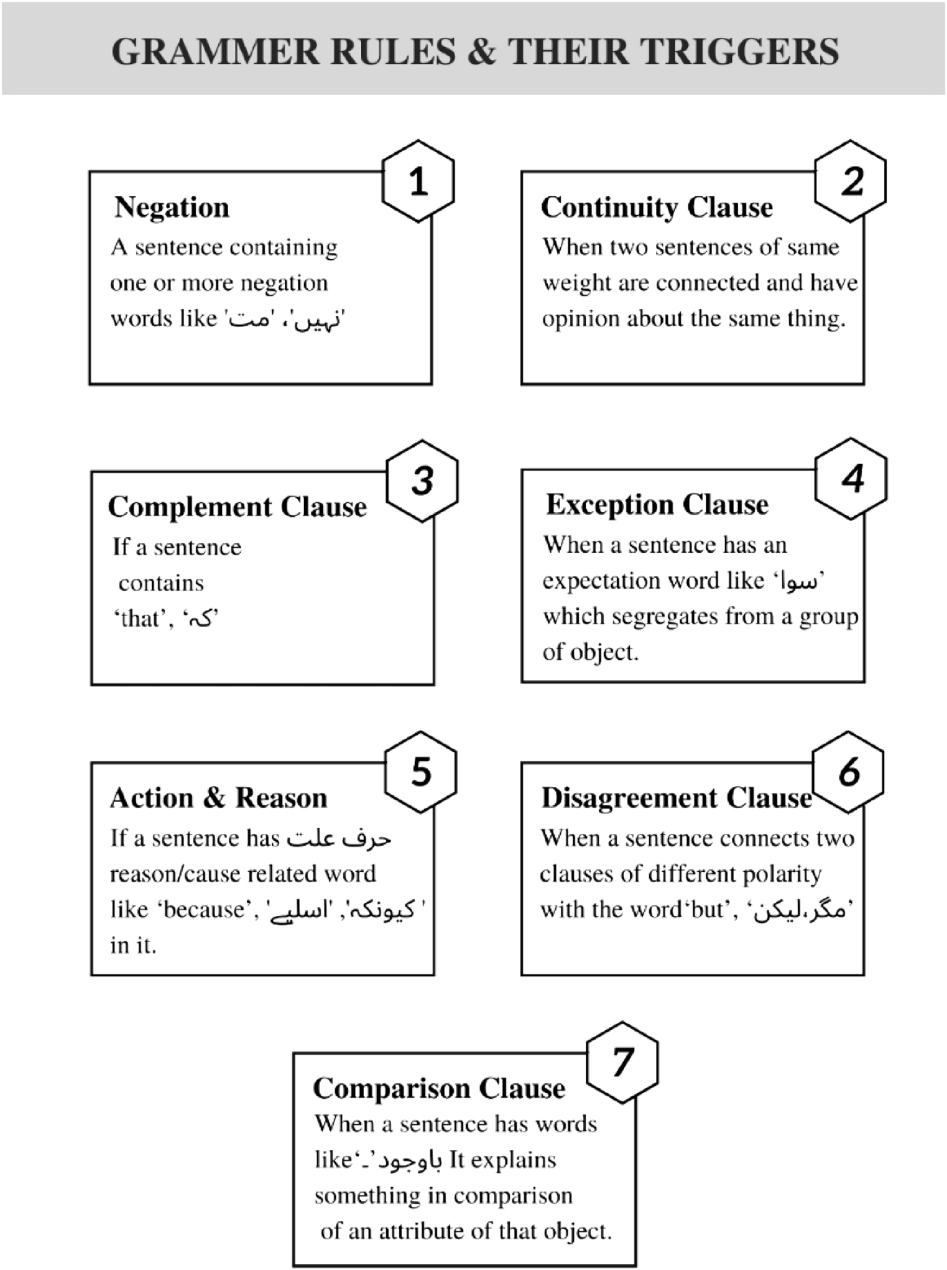
List of Urdu grammar-based rules and their trigger words and events.

As illustrated in Fig. 6, several grammatical rules have been identified that contribute to a sentence's polarity alteration. This section also discusses the grammatical rules in detail, when they are triggered, and how the polarity is determined in the event of a trigger.

#### Negation

***Trigger***: A sentence containing one or more negation words like 'نہیں'، 'مت'.

***Action***: Overall polarity of the sentence is changed based on the sentence or concept with which negation is being used. If a negative concept is negated, then the polarity of the sentence is positive, and if a positive token is negated, then the polarity of the sentence is negative. For example, یہ کتاب مجھے پسند نہیں ہے, ‘I don’t like this book’ has negative polarity. On the other hand, ‘میں نے یہ کتاب پڑھی ہے، اسے خرید نے سے کترایئے مت۔’, has overall positive polarity.

#### Continuing clause (حروف وصل)

***Trigger***: When two sentences of the same weight are connected and have an opinion about the same thing. The word 'and' 'اور' usually connect two sentences having a continuing relationship.

***Action***: If one of the sentences has positive polarity, the other part of the sentence also has positive polarity, resulting in a positive overall polarity for that sentence. If one of the sentences has negative polarity, the other part of the sentence also has negative polarity, resulting in a negative overall polarity for that sentence. For example, this mobile has low battery timing, and its camera is also not good. 'اس موبائل کی بیٹری کی میعاد کم ہے اور اس کا کیمرہ بھی اچھا نہیں ہے' so this sentence has overall negative polarity.

#### Complement clause

***Trigger***: If a sentence contains ‘that’, ‘کہ’.

***Action***: A sentence containing ‘کہ’, and ‘that’ is divided into two parts, and the polarity of the first part is considered the overall polarity of the sentence. For example: Good thing about Samsung mobile is that it has good camera and battery timing, ‘سامسنگ موبائل کی اچھی بات یہ ہے کہ اس کا کیمرہ اور بیٹری کا معیادِاستعمال اچھی ہے’.

#### Exception clause

***Trigger***: When a sentence has an expectation word like ‘سوا', which segregates an object from a group of objects.

***Action***: In cases when two clauses have an opinion about a group of objects and an exception clause is used for separation in two clauses. In such cases, the polarity for a single entity that is an exception from another is found in one clause that is before the exception word, and polarity of the group can be found in the other. For example, Except for this mobile phone, which is better, all other mobiles do not work properly. 'یہ موبائل بہتر ہے، اس کے سوا تمام موبال ٹیھک کام نہیں کرتے''. In this sentence first clause has a positive polarity and the other clause has negative polarity. Overall polarity usually depends on the polarity of the first clause.

#### Action and reason clause

***Trigger***: If a sentence has حرف علت reason/cause related word like ‘because’, 'کیونکہ', 'اسلیے ' in it. The sentences that contain opinion/compliment anything in the first clause and then second clause starting with a word like 'کیونکہ' has an explanation of the reason for opinion or complement.

***Action***: Polarity in the case of the action and reason clause is determined with respect to the polarity of the first clause as it explains opinion about anything in the first part and then gives a reason for that in the second clause of the sentence. For example: 'مجھے یہ کرسی پسند ہے کیونکہ یہ مضبوط ہے' I like this chair because this is durable. The overall polarity of this sentence is positive, which is extracted from the polarity of the first clause of the sentence.

Proposition Clause (حروف جار).

#### Disagreement clause (حروف استدراک)

***Trigger***: When a sentence connects two clauses of different polarity with the word ‘but’, ‘مگر،لیکن’.

***Action***: The first part of the sentence has some disagreement which is then clarified in the second part of that sentence which is after ‘مگر،لیکن’۔ So if the first part of the sentence has negative polarity, the second part would have clarification of the disagreement in it and would have positive polarity. The overall polarity of the sentence having a disagreement clause is in the second clause that is after the word 'مگر،لیکن'. So, if the sentence has negative polarity in the second clause, the sentence has negative polarity. On the other hand, if the second clause has positive polarity sentence has positive polarity. For example, this book is expensive, but I like the quality of the book, 'یہ کتاب مہنگی ہے مگر مجھے اس کا معیار پسند ہے', as the second clause has positive polarity, so the polarity of the sentence is positive.

#### Comparison clause

***Trigger***: When the sentence has words like ‘باوجود’۔, It explains something in comparison to an attribute of that object.

***Action***: Sentence with comparison to an attribute of an object has polarity based on clause after comparison word. For example: Despite high prices, the quality of this mobile is very low, ' زیادہ قیمت کی باوجود اس موبائل کا معیار بہت کم ہے' ۔ This sentence has negative polarity as clause after 'باوجود has negative polarity.

## A dependency rules-based Sa framework

**Figure 7:**
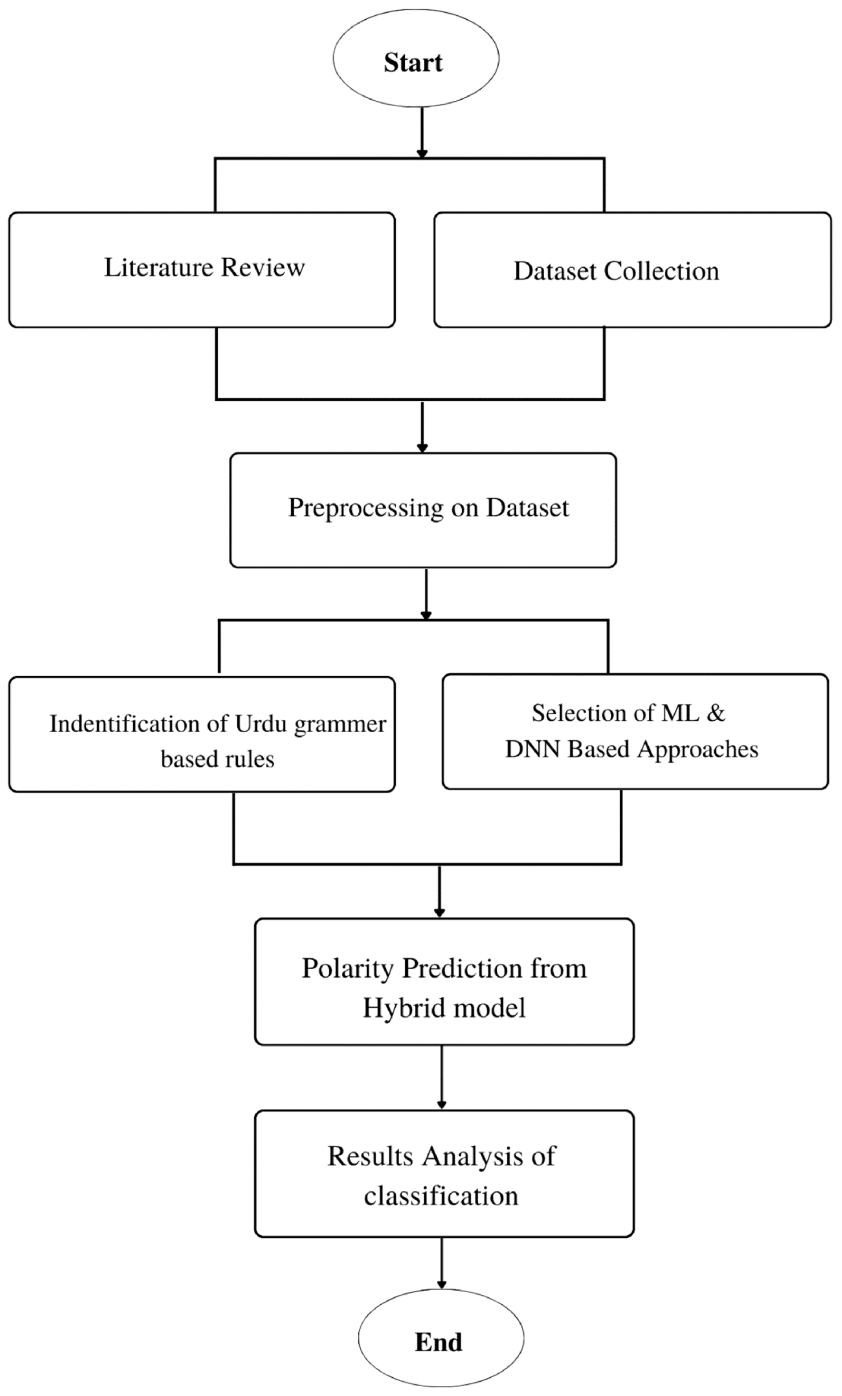
Our proposed research methodology model for SA is based on Urdu grammatical dependency-based rules model.

Here, the grammatical dependency rules for Urdu were combined with ML models, such as SVM, LR, and DNN models like LSTM and CNN. The primary goal of this integrated approach is to accurately classify Urdu sentences whose polarity or sentiment cannot be effectively determined using conventional word-based methods that solely rely on positive or negative words. By incorporating the grammatical dependency rules, which capture the interdependencies and relationships between words within a sentence, into the ML models, this research aimed to enhance the sentiment analysis process. This integration enables the framework to capture subtle nuances in sentiment that may go unnoticed by traditional word-based techniques. The central focus of this approach is to accurately classify sentences that demonstrate complex sentiment patterns, where determining polarity solely based on individual positive or negative words is challenging. By combining the linguistic knowledge embedded in the grammatical dependency rules with the predictive power of the ML models, the framework becomes more proficient in handling these intricate cases effectively. The steps of the proposed hybrid framework are depicted in Fig. 7 and are discussed here.

### Data preprocessing

Tokenisation and normalisation techniques are used to pre-process the corpus. The sentences were stripped of numbers and punctuation. The sentences were already manually tagged while creating the dataset, and the polarity per word was refined further, with zero polarity assigned to words that didn’t appear in the lexicon. In the end, a dependency tree was generated to identify the dependency tree for a sentence. All of this was done with the urduhack python package for the Urdu language^32^ and^33^. The recommended dependency-based rules classifier is fed the dependency tree and assigned polarities. The presented classifier is fed a dependency tree as well as the assigned polarities.

### Polarity prediction algorithm

To classify unseen sentences, the proposed framework incorporates the language’s dependency-based rules into the deep learning architecture. Below is the pseudocode for the proposed method:

**Figure:**
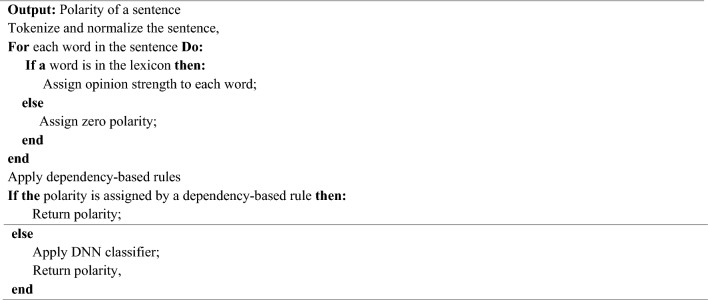
Algorithm: Polarity Prediction

#### Long short-term memory (LSTM)

**Figure 8:**
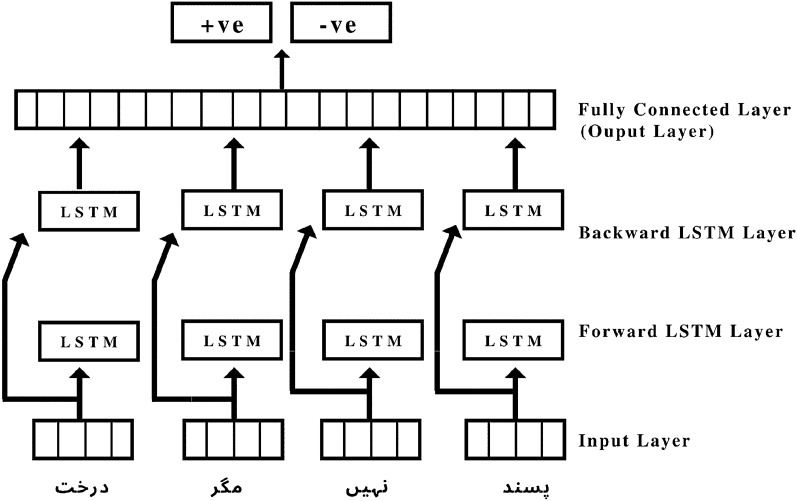
BI-LSTM Deep Learning Model for classification of sentiments from Urdu sentences of the dataset.

As illustrated in Fig. 8, the proposed LSTM configuration includes input layers from which parsed Urdu sentences are passed to the model. The following two layers of the model are stacked bidirectional LSTM layers with 128 and 64 cells, respectively. Following these layers are a dropout layer and a dense layer with two neurons and softmax activation. The model’s final layer is a completely connected output layer that determines the polarity of the sentences passed to it from the input layer^5^.

#### Convolutional neural network (CNN)

**Figure 9:**
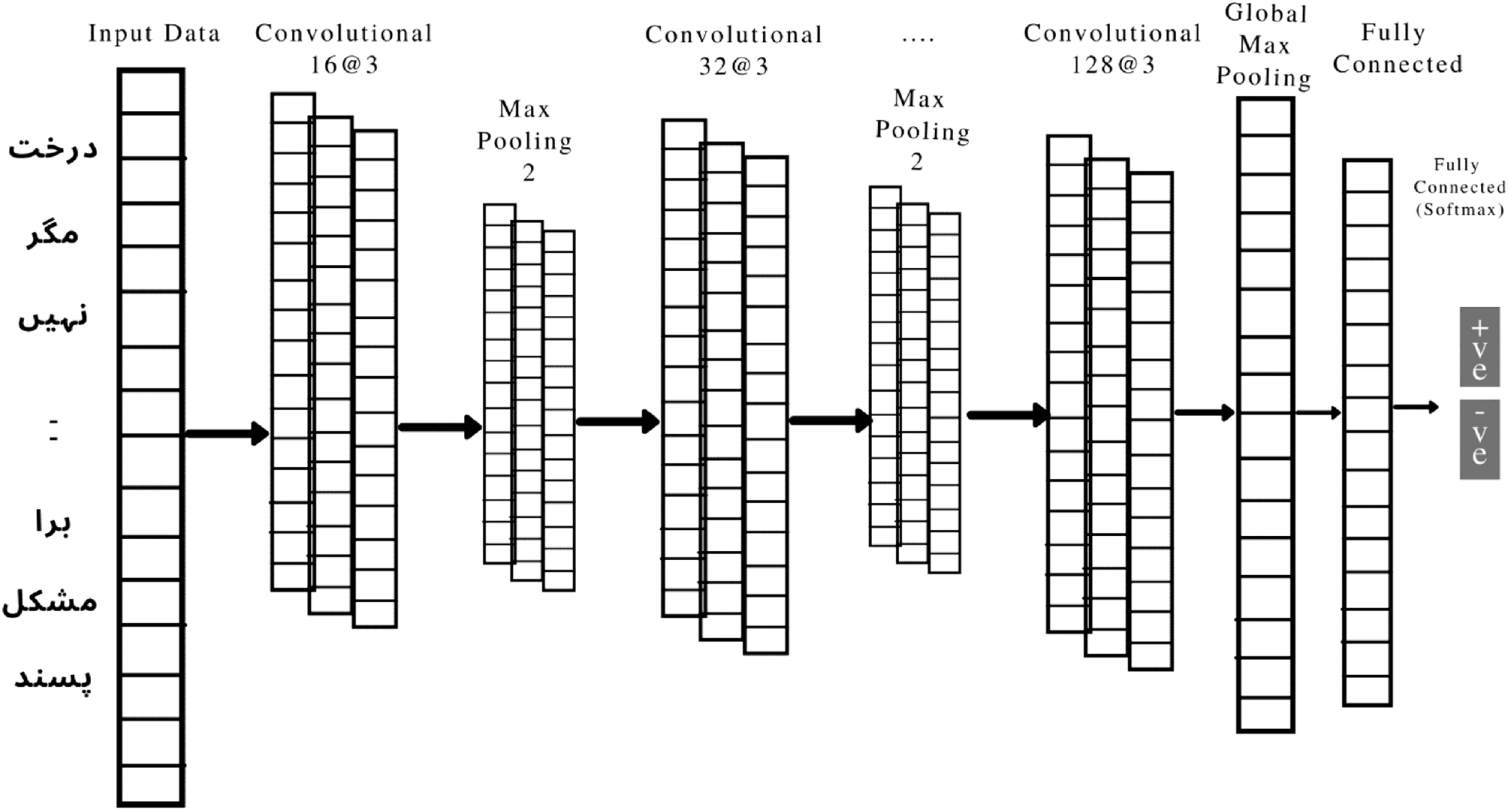
CNN DL Model for classification of sentiments from Urdu sentences of the dataset.

The CNN model that was used in this experimental study is depicted in Fig. 9. The model was trained using grammatical rules for detecting polarity in the form of negative or positive reviews of people from a set of reviews on films, products, and politics.

It is common for the rule-based approach to use positive polarity to classify sentences when word polarity is not available due to the small Urdu lexicon. SVM, LR, and MLP classifiers have also been used as a baseline to compare the performance of proposed approach. In order to train and validate the DNN architectures, the TensorFlow library and Google Colab Pro GPU were used. Backpropagation has been utilised for training the models for 100 epochs, and the Adam optimiser was used to minimise the categorical cross-entropy loss. As part of the hybrid framework, the rule-based approach's unclassified sentences were transformed into 200-dimensional fastText word embedding and fed into deep learning classifiers.

## Dataset

For previous dataset^17^, the chosen textual contents generated through video transcription was utilized. The dataset consisted of speakers aged between 20 and 40 years and included videos with an average duration ranging from 3 to 8 min. It was categorized into three distinct genres: film reviews, political commentary, and product reviews. The training set, which comprised 70% of the data, was used to train the models, while the test set, accounting for 30% of the dataset, was employed for evaluating and reporting the results.

### Urdu movie review dataset

The Urdu movie review dataset contains 3000 reviews provided by various users, covering a wide range of films. It consists of 15,000 positive reviews and 15,000 negative reviews.

### Political review dataset

The political review dataset comprises 4000 reviews, including 2000 positive reviews and 2000 negative reviews.

### Product review dataset

The Urdu product review dataset comprises 2000 reviews from different users, with 1000 positive reviews and 1,000 negative reviews.

### Availability of data and materials

The dataset was publicly available on GitHub (https://github.com/uroobasehar/datasethybriddependencybasedmodel) for researchers to utilize in further experiments related to Urdu sentiment analysis models^32^.

## Results and analysis

Three datasets were used for conducting the experiments. The results of both hybrid models and the LSTM and CNN models are summarised in Table 1, along with comparisons to other models and techniques proposed by various researchers in the literature. An accuracy of approximately 74.69% using SVM was obtained, while the precision, recall, and F measures were 0.74, 0.73, and 0.74, respectively. On the movies review dataset, an accuracy of 72.53% was obtained using an LR model, with precision (P), recall (R), and f-measure (F) values of 0.72, 0.71, and 0.72, respectively. Similarly, MLP alone provided an accuracy of approximately 73.92%, as well as precision, recall, and f-measure values of approximately 0.73,0.72, and 0.73, respectively. When the proposed dependency-based rules are applied, a significant improvement was observed in the accuracy of classifying Urdu sentences from the movie reviews dataset. As illustrated in Table 1, When dependency-based rules are used alone, accuracy improves by approximately 6–7%, as an accuracy of approximately 80.56 percent was acquired along with P, R, and F values of approximately 0.80, 0.79, and 0.80, respectively. As a result of the experiments, a noticeable improvement in classification accuracy was observed when using DNN models such as CNN and LSTM.

**Table 1:** Summary of results of movie reviews.

Classifier	Accuracy %	Precision	Recall	F-measure
Mukhtar et al.^27^	72.53	0.72	0.71	0.72
Ghulam et al.^34^	0.97	0.9287	0.94	0.95
Khan et al.^29^	74	0.74	0.73	0.74
SVM	74.69	0.74	0.73	0.74
Logistic Regression	72.53	0.72	0.71	0.72
MLP	73.92	0.73	0.72	0.73
Dependency Based Rules	80.56	0.80	0.79	0.80
CNN	82.61	0.82	0.81	0.82
LSTM	83.92	0.83	0.82	0.83
Hybrid 1: CNN + Dependency-based rules	87.93	0.87	0.86	0.87
Hybrid 2: LSTM + Dependency-based rules	89.75	0.89	0.88	0.89

Both the hybrid models, a combination of LSTM with dependency-based rules and a combination of CNN with dependency-based rules, have shown an improvement in accuracy of about 15–17% from the state-of-the-art models. In comparison to both hybrid approaches, hybrid 2, which is a combination of LSTM and dependency-based rules, performed best among all other models by achieving an accuracy of 89.75% and P, R, and F of 0.89, 0.88, and 0.89, respectively.

An ablation study was also performed in order to know the way each part works in isolation. Tables 2, 3, and 4 report the outcome of ablation research on the movie, hotel as well as product review corpora, respectively. Experimental results show that the exceptional clause achieved better accuracy in all review datasets in comparison to various other rules. The disagreement clause achieved the lowest performance compared to other rules.

**Table 2:** Ablation study using movie reviews Dataset.

Classifier	Accuracy %	Precision	Recall	F-measure
Negation	52.65	0.52	0.51	0.52
Conjunction clause	51.36	0.51	0.50	0.51
Complement clause	53.3	0.53	0.52	0.53
Exceptional clause	52.42	0.52	0.51	0.52
Exceptional clause	56.79	0.56	0.55	0.56
Action and reason clause	55.41	0.55	0.54	0.55
Disagreement clause	51.39	0.51	0.50	0.51
Comparison clause	52.66	0.52	0.51	0.52

**Table 3:** Summary of results of political reviews.

Classifier	Accuracy %	Precision	Recall	F-measure
Mukhtar et al.^27^	74.53	0.74	0.73	0.74
Ghulam et al.^34^	0.97	0.9287	0.94	0.95
Khan et al.^29^	76.91	0.76	0.75	0.76
SVM	79.78	0.79	0.78	0.79
Logistic Regression	81.37	0.81	0.80	0.81
MLP	78.54	0.78	0.77	0.78
Dependency Based Rules	85.46	0.85	0.84	0.85
CNN	86.91	0.86	0.85	0.86
LSTM	87.46	0.87	0.86	0.87
Hybrid 1: CNN + Dependency-based rules	91.59	0.91	0.90	0.91
Hybrid 1: LSTM + Dependency-based rules	93.06	0.93	0.92	0.93

**Table 4:** Ablation study using political reviews dataset.

Classifier	Accuracy %	Precision	Recall	F-measure
Negation	53.61	0.53	0.52	0.53
Conjunction Clause	54.29	0.54	0.53	0.54
Complement clause	55.32	0.55	0.54	0.55
Exceptional clause	52.63	0.52	0.51	0.52
Exceptional clause	51.8	0.51	0.50	0.51
Action and reason clause	52.93	0.52	0.51	0.52
Disagreement clause	54.67	0.54	0.53	0.54
Comparison clause	53.59	0.53	0.52	0.53

Experiments on the political review dataset are reported in Table 4. Hybrid models outperformed all other approaches in terms of accuracy, with a score of 93.05%, P, R, and F of 0.93, 0.92, and 0.93, respectively. Similarly, for the product review dataset, hybrid models outperform the other models (Table 4).

Table 5 summarises the results of the experiments carried out to compute polarity using the proposed hybrid models for various sentences from the datasets. It can be seen that complex sentences with multiple clauses and phrases that have different polarities due to grammatical aspects hidden within those sentences are correctly classified. It is because of this that the hybrid approach takes into account the language's dependency rule.

**Table 5:** Summary of results.

Example of sentence	Sentiment polarity
میں نے ایٹم بم بنایا ھے ۔۔۔۔او بھائی ایٹم بمب کوٹ لکھپت والی اتفاق فیکٹری میں نہیں بنتا۔ایٹم بم کہوٹہ کی ایٹمی۔	Positive
چندے سے انقلاب اور عمران خان وزیر اعظم نہیں بن سکتے	Negative
سرچ انجن گوگل کے نائب صدر نے فضا میں ، 130,000 فٹ کی بلندی پر چھلانگ لگا کر عالمی ریکارڈ قائم کرلیا۔ چھلانگ کی۔۔۔	Positive
ابن ڈیزل ہواُکرے کوئی میرے درد کی دوا کرے کوئی	Negative

**Figure 10:**
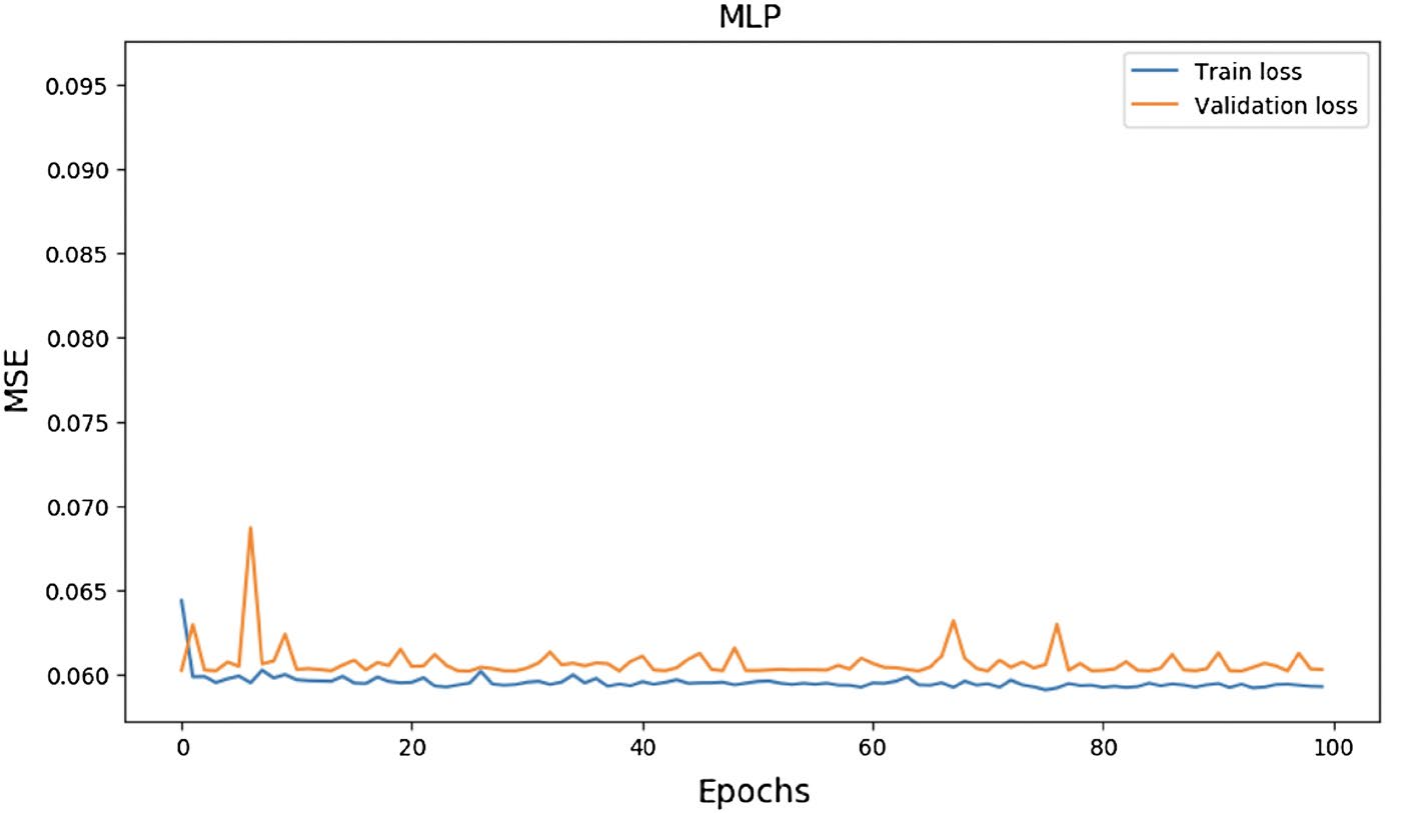
Train and validation loss for MLP Model over 100 epochs.

**Figure 11:**
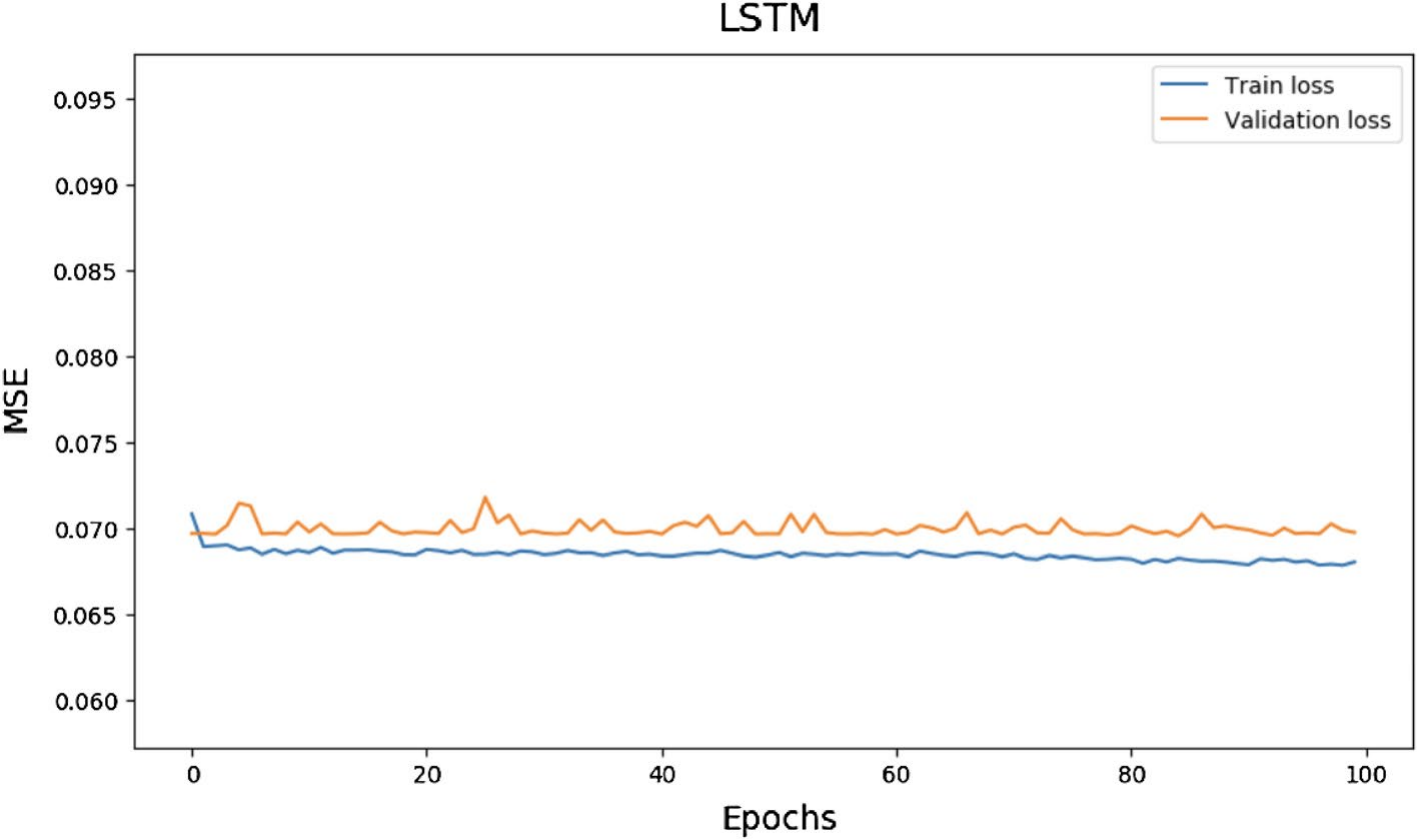
Train and validation loss for LSTM Model over 100 epochs.

Figure 10, 11, and 12 demonstrate the evolution of learning curves, which provide insight into the behaviour of various models. The learning curve is smoothing out over time.

**Figure 12:**
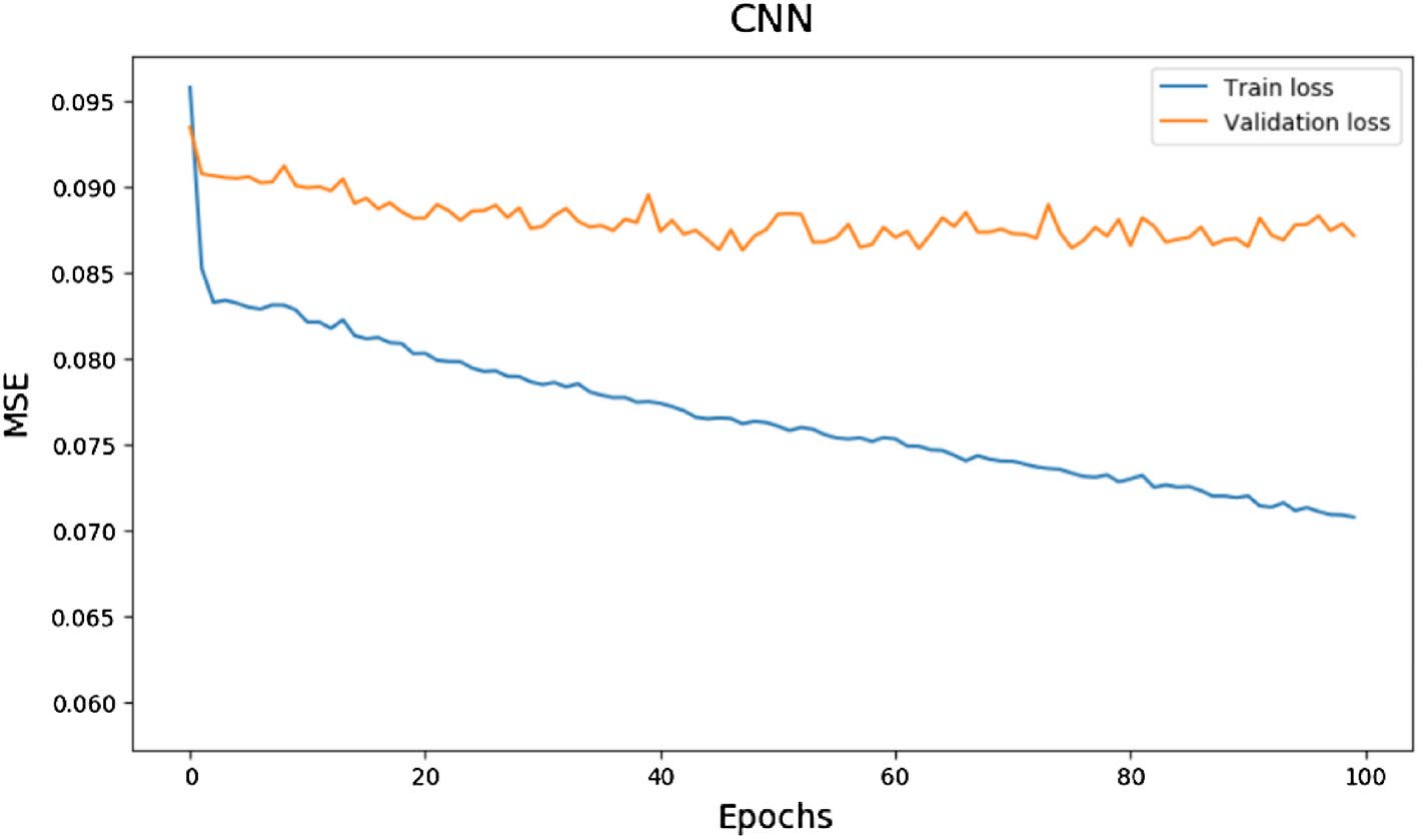
Train and validation loss for CNN Model over 100 epochs.

## Conclusion and future work

Digital media, as an integral part of our daily lives, plays a crucial role in the distribution and generation of massive amounts of data daily, containing the perspectives of diverse people from diverse regions of the world on a variety of subjects and issues. Reviewing products and services and leaving comments on items sold on e-commerce sites has become a widespread trend that almost everyone is now following. With this deluge of data generated daily, the need for data processing and analysis becomes apparent in order to leverage the data to enhance product and service quality. Over the last decade, researchers have actively contributed to the body of knowledge regarding SA in a variety of languages spoken by people worldwide. Urdu SA continues to require researchers' attention in order to develop effective and efficient models for detecting the polarity of sentiments expressed in Urdu sentences shared by people on the internet about various products and services they use in their daily lives. In this study, we propose a hybrid framework for detecting the polarity of sentiments in Urdu using multiple deep neural network approaches and dependency-based Urdu language grammatical rules. This work is a continuation of previous work^8^, in which multimodal SA was used. Three distinct datasets were used in these experiments: movie reviews, product reviews, and political reviews. Results were reported using SVM, Logistic Regression, Multilayer Perceptron (MLP), and Decision Tree (DL) models, as well as DL models combined with dependency-based rules for improved prediction. Experimental results demonstrate that the proposed hybrid approach outperforms state-of-the-art SA methods by nearly 10%.

In the future, it is recommended to address the issue of unclassified sentences by expanding our lexicon and to investigate the generalisation capability of hybrid framework by utilising additional challenging corpora from a variety of different applications, including emotion-sensitive companions. It is intended to optimise the prediction model by using the hyperparameter optimisation technique suggested in^35^. Further, it is intended to investigate multimodal datasets with language dependency rules.

## Data Availability

The dataset was publicly available on GitHub (https://github.com/uroobasehar/datasethybriddependencybasedmodel) for researchers to utilize in further experiments related to Urdu sentiment analysis models^32^.
